# Critical Roles of the Direct GABAergic Pallido-cortical Pathway in Controlling Absence Seizures

**DOI:** 10.1371/journal.pcbi.1004539

**Published:** 2015-10-23

**Authors:** Mingming Chen, Daqing Guo, Min Li, Tao Ma, Shengdun Wu, Jingling Ma, Yan Cui, Yang Xia, Peng Xu, Dezhong Yao

**Affiliations:** 1 Key Laboratory for NeuroInformation of Ministry of Education, School of Life Science and Technology, University of Electronic Science and Technology of China, Chengdu 610054, People’s Republic of China; 2 Center for Information in Medicine, University of Electronic Science and Technology of China, Chengdu 610054, People’s Republic of China; Universitat Pompeu Fabra, SPAIN

## Abstract

The basal ganglia (BG), serving as an intermediate bridge between the cerebral cortex and thalamus, are believed to play crucial roles in controlling absence seizure activities generated by the pathological corticothalamic system. Inspired by recent experiments, here we systematically investigate the contribution of a novel identified GABAergic pallido-cortical pathway, projecting from the globus pallidus externa (GPe) in the BG to the cerebral cortex, to the control of absence seizures. By computational modelling, we find that both increasing the activation of GPe neurons and enhancing the coupling strength of the inhibitory pallido-cortical pathway can suppress the bilaterally synchronous 2–4 Hz spike and wave discharges (SWDs) during absence seizures. Appropriate tuning of several GPe-related pathways may also trigger the SWD suppression, through modulating the activation level of GPe neurons. Furthermore, we show that the previously discovered bidirectional control of absence seizures due to the competition between other two BG output pathways also exists in our established model. Importantly, such bidirectional control is shaped by the coupling strength of this direct GABAergic pallido-cortical pathway. Our work suggests that the novel identified pallido-cortical pathway has a functional role in controlling absence seizures and the presented results might provide testable hypotheses for future experimental studies.

## Author Summary

One prevailing viewpoint on the genesis of absence seizures is suggested to involve the pathological interactions within the corticothalamic system. As intriguing deep nuclei of brain, the basal ganglia are reported to play crucial roles in controlling absence seizures through multiple nigro-thalamic pathways. Beside these nigro-thalamic pathways, recent experiments have identified a new direct GABAergic pallido-cortical pathway projecting from the globus pallidus externa (GPe) to the cerebral cortex. Does this novel identified inhibitory pallido-cortical pathway also participate in regulating absence seizures? By computational modelling, we demonstrated that this inhibitory pallido-cortical pathway indeed participates in the control of absence seizures. Specifically, both activating the GPe neurons and enhancing the strength of this pathway significantly inhibit the typical 2–4 Hz spike and wave discharges (SWDs) during absence seizures. Further investigation showed that appropriate tuning of several GPe-related pathways may trigger the SWD suppression by activating GPe neurons, and also the strength of this inhibitory pallido-cortical pathway may regulate the so-called bidirectional control of absence seizures caused by direct nigro-thalamic pathways. These results highlight the functional roles of the GABAergic pallido-cortical pathway in controlling absence seizures and have important physiological implications in the treatment of absence epilepsy.

## Introduction

Epilepsy is a paroxysmal behavior caused by abnormal, excessive or hypersynchronous discharges of neurons in the brain [1, 2]. Absence epilepsy (AE) is a subtype of idiopathic generalized epilepsy and mainly occurs in the childhood years [3, 4]. Experimental studies showed that a typical attack of absence seizures commonly causes a sudden onset and offset of spike-wave activities, accompanying with temporary loss of consciousness. As an electrophysiological hallmark of AE, the bilaterally synchronous spike and wave discharges (SWDs) with a slow frequency around 2–4 Hz is often observed on the electroencephalogram (EEG) of absence epileptic patients [5]. So far, a large number of studies have been performed to investigate the generation mechanisms of typical SWDs during absence seizures [6–8]. There is accumulating evidence that such type of SWDs is highly associated with the abnormal interactions between cerebral cortex and thalamus, and appropriately regulating the pathological corticothalamic system may control absence seizures [9–14].

As key deep nuclei of brain, the basal ganglia (BG) consist of a collection of important subcortical structures, such as striatum, substantia nigra, globus pallidus and subthalamic nucleus [15]. In addition to the direct coupling, anatomical studies have revealed that the cerebral cortex and thalamus also communicate indirectly via the BG [15, 16]. In particular, it has been found that the BG send output signals to thalamus through multiple direct and indirect inhibitory nigro-thalamic pathways. It is thus proposed that the BG may participate in the control of absence seizures through these nigro-thalamic pathways. Several existing experimental and computational results support this hypothesis [17–20]. For instance, previous experimental studies have indicated that pharmacological inactivation of the substantia nigra pars reticulata (SNr) may considerably suppress the generation of 2–4 Hz SWDs via the indirect nigro-thalamic pathway relaying at superior colliculus [17–19]. Moreover, using a biophysically based mean-field model, we have demonstrated that the BG can control and modulate typical absence seizure activities in a bidirectional manner, due to the competition between two direct inhibitory nigro-thalamic pathways [21].

On the other hand, outputs from the BG to the cerebral cortex are traditionally thought to be relayed through the thalamus. This opinion, however, has been challenged by recent experimental observations [22, 23]. For example, lesions of the globus pallidus externa (GPe) in the BG produce significant increases in wakefulness [24], but lesions of the thalamus have a minimal effect on overall sleep-wake patterns [25]. Given that the thalamus is the putative output relay for the BG, the thalamic lesion should also influence the sleep-wake patterns greatly. More importantly, combining both retrograde and anterograde tracing techniques, recent experiments have identified the existence of a direct efferent output from the GPe to both excitatory pyramidal neurons and inhibitory interneurons in the cerebral cortex [22, 23]. Such pallido-cortical projection is found to be mediated by GABA_A_ receptors and its postsynaptic targets are primarily distributed in the frontal cortex. Because several subregions of the frontal cortex are reported to be highly associated with AE [26, 27], it is thus interesting to know whether this inhibitory pallido-cortical pathway can provide an alternative mechanism to regulate typical absence seizure activities. Thus far, the direct experimental evidence to support this function is still lacking, and computational modelling might outline several possible roles of this GABA_A_-mediated pathway in the control and modulation of absence seizures.

Here we approach the aforementioned issue theoretically using a modified basal ganglia-corticothalamic (BGCT) network model [21, 28–30]. Our main finding is that both increasing the activation of GPe neurons and enhancing the coupling strength of the GABAergic pallido-cortical pathway can significantly suppress the typical 2–4 Hz SWDs during absence seizures. Several GPe-related pathways, which directly and indirectly modulate the activation level of GPe neurons, are also found to play important roles and suitably tuning their coupling strengths may trigger the SWD suppression. Further explorations confirm that the recently identified bidirectional control of absence seizures by the BG also exists in our modified model [21], which is shaped by the strength of the pallido-cortical pathway. Together, these results demonstrate an intriguing hypothesis that the BG may regulate absence seizures through the direct inhibitory projection from the GPe to the cerebral cortex, and might provide meaningful insights into the pathogenesis and treatment of AE.

## Materials and Methods

### Network structure

**Fig 1:**
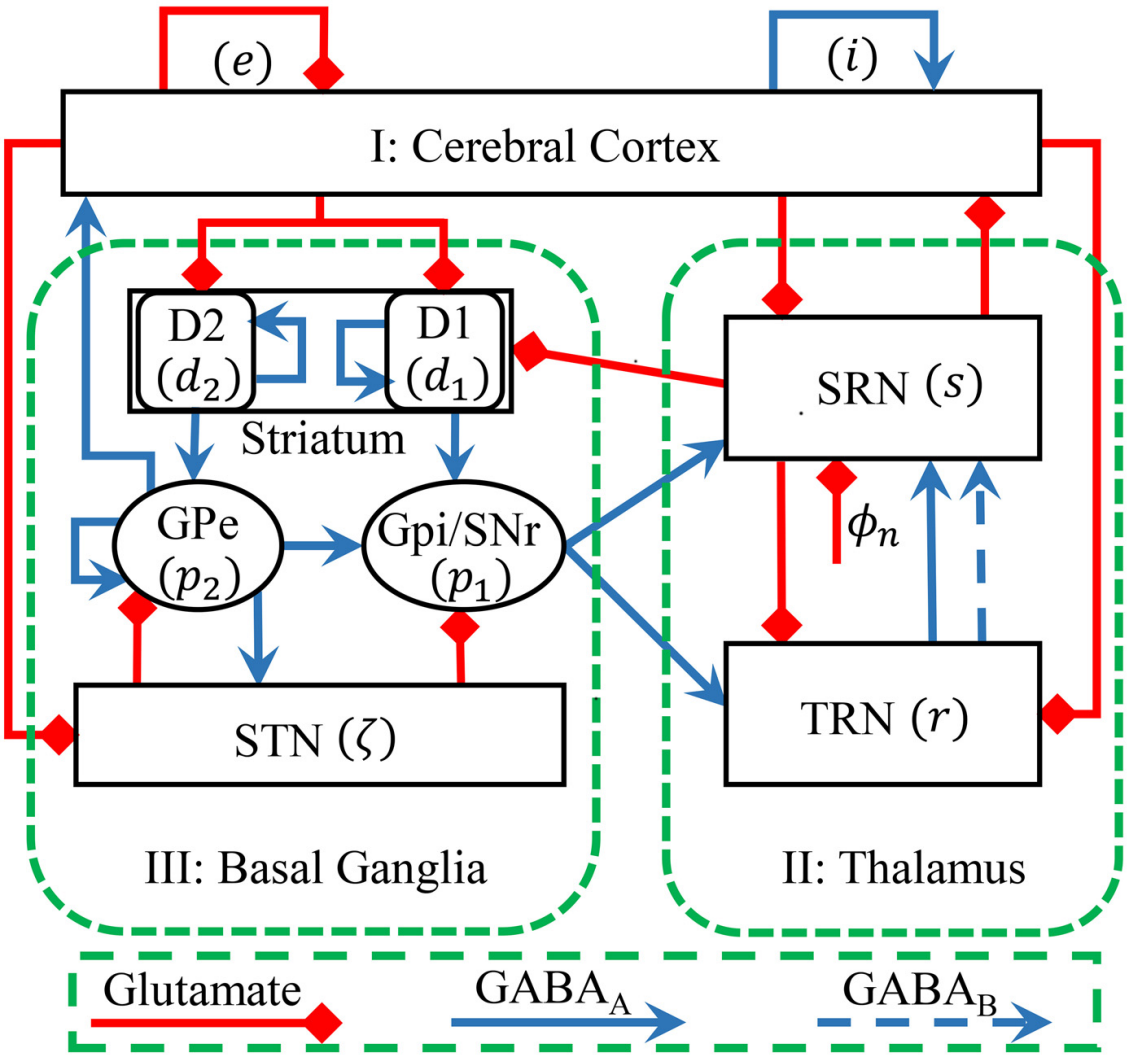
Framework of the basal ganglia-corticothalamic (BGCT) network used in this work. The BGCT network contains three components: (I) the cerebral cortex, (II) the thalamus and (III) the basal ganglia. Neural populations include: *e* = excitatory pyramidal neurons, *i* = inhibitory interneurons, *s* = SRN, *r* = TRN, *d*_1_ = striatal D1 neurons, *d*_2_ = striatal D2 neurons, *p*_1_ = SNr/GPi, *p*_2_ = GPe and ζ = STN. Parameter *ϕ*_*n*_ denotes the non-specific external inputs to SRN. Excitatory projections are mediated by glutamate, which are shown by the red lines with square heads. Inhibitory projections are mediated by GABA_A_ and GABA_B_, which are represented by the solid and dashed blue lines with arrow heads, respectively. Compared with the BGCT networks developed in previous studies, a new efferent pathway representing direct connection from the GPe to the cerebral cortex is incorporated in our current BGCT model.

In recent studies, we have established a biophysically based BGCT model to investigate the bidirectional control of absence seizures by the basal ganglia through two direct inhibitory nigro-thalamic pathways [21, 30]. To explore the underlying functional roles of the novel identified inhibitory pallido-cortical pathway in controlling absence seizures, we implemented a modified version of this BGCT model by incorporating a new efferent pathway representing direct connection from the GPe to the cerebral cortex. The network architecture of our current BGCT model is derived from the anatomical data of rodents, which is schematically illustrated in Fig 1. Briefly, the model comprises nine neural populations: excitatory pyramidal neurons, inhibitory interneurons, specific relay nuclei (SRN), thalamic reticular nucleus (TRN), striatal D1 neurons, striatal D2 neurons, substantia nigra reticulata (SNr), globus pallidus externa (GPe) and subthalamic nucleus (STN). Similar to previous modelling studies [21, 28–30], the globus pallidus internal (GPi) segment is regarded as a single structure with SNr in our model, because they have the similar properties in both neural function and anatomical connectivity [28, 29]. For simplicity, we use mathematical notations *a* ∈ *A* = {*e*, *i*, *s*, *r*, *d*_1_, *d*_2_, *p*_1_, *p*_2_, *ζ*} to indicate above neural populations. Following previous work [21, 28–30], a non-specific external input *ϕ*_*n*_ is injected to the SRN to model the sensory input. As shown in Fig 1, three types of neural projections are considered in our modified BGCT model. The red lines with square heads represent the excitatory projections mediated by glutamate. The blue solid and dashed lines with arrow heads denote the GABA_A_- and GABA_B_-mediated inhibitory projections, respectively. Compared with other types of projections, a transmission delay is introduced to the GABA_B_-mediated inhibitory pathway projecting from TRN to SRN to mimic its relatively slow synaptic kinetics (see below).

### Mean-field model

We use the mean-field model developed by Robinson and his colleagues to simulate the dynamics of neural populations [28, 29, 31–34]. This model was proposed to describe the macroscopic dynamics of neural populations in an effective way with low computational cost. In this mean-field model, the dynamics of each neural population is determined by three key variables: the mean membrane potential *V*_*a*_, the mean firing rate *Q*_*a*_ and the presynaptic activity *ϕ*_*a*_. The relationship between the mean firing rate and the mean membrane potential satisfies a sigmoid function defined as [28–36]:

$$\begin{array}{c} Q_{a}\left(r,t\right)\equiv F\left[V_{a}\left(r,t\right)\right]=\frac{Q_{a}^{max}}{1+\text{exp}[-\frac{\pi }{\sqrt{3}}\frac{\left(V_{a}\left(r,t\right)-\theta_{a}\right)}{\sigma }]}, \end{array}$$ (1)

where subscripts *a* ∈ *A* = {*e*,*i*,*r*,*s*,*d*_1_,*d*_2_,*p*_1_,*p*_2_,*ζ*} indicate different neural populations, **r** denotes the spatial position, and *θ*_*a*_ is the mean firing threshold. Parameters $Q_{a}^{max}$ and *σ* refer to the maximum and standard deviation of the firing rate, respectively. For each neural population, the sigmoid-type function in Eq (1) restricts the mean firing rate *Q*_*a*_ in the range of 0 and $Q_{a}^{max}$, ensuring its physiological reasonableness. The fluctuations of the mean membrane potential *V*_*a*_ at the position **r** are induced by the filtered incoming postsynaptic potentials from other neural populations in the dendrites, which are modelled as [28–36]:

$$\begin{array}{c} D_{\alpha \beta }V_{a}\left(r,t\right)=\sum_{b\in A}v_{ab}\cdot \phi_{b}\left(r,t\right), \end{array}$$ (2)

$$\begin{array}{c} D_{\alpha \beta }=\frac{1}{\alpha \beta }\left[\frac{\partial^{2}}{\partial t^{2}}+\left(\alpha +\beta \right)\frac{\partial }{\partial t}+\alpha \beta \right]. \end{array}$$ (3)

Here the differential operator *D*_*αβ*_ represents the synaptic and dendritic filtering of incoming signals. *α* and *β* indicate the inverse decay and rise time constants of cell-body potential caused by received signals, respectively. *v*_*ab*_ represents the coupling strength from the neural population of type *b* to type *a*. *ϕ*_*b*_(**r**,*t*) is the presynaptic activity of the neural population of type *b*. For the GABA_B_-mediated inhibitory projection, a delay parameter *τ* is introduced to its incoming pulse rate (i.e., *ϕ*_*r*_(**r**,*t* − *τ*)) to simulate its slow synaptic kinetics [21, 35]. Besides, we do not consider the transmission delay for other types of neural projections in the current work. The above mathematical treatment leads to a delay differential equation in the final description of our modified BGCT model (see S1 Text).

In the cerebral cortex, the outward propagation of excitatory firing rates *Q*_*e*_ along the cortical surface obeys the damped wave equation [28–36]:

$$\begin{array}{c} \frac{1}{\gamma_{e}^{2}}\left[\frac{\partial^{2}}{\partial t^{2}}+2\gamma_{e}\frac{\partial }{\partial t}+\gamma_{e}^{2}-v_{e}^{2}\nabla^{2}\right]\phi_{e}\left(r,t\right)=Q_{e}\left(r,t\right), \end{array}$$ (4)

Here *ϕ*_*e*_ represents the cortical excitatory axonal field and ∇^2^ denotes the Laplacian operator (the second spatial derivative). The parameter *γ*_*e*_ = *v*_*e*_/*r*_*e*_ governs the temporal damping rate of pulses, where *v*_*e*_ and *r*_*e*_ are the conduction velocity and characteristic range of axons of excitatory neurons, respectively. Besides excitatory pyramidal neurons, the axons of all other neural populations are believed to be too short to support wave propagation on the respective scales in our model, indicating that *ϕ*_*k*_ = *F*[*V*_*k*_], (*k* = *i*,*s*,*r*,*d*_1_,*d*_2_,*p*_1_,*p*_2_,*ζ*). Because AE is a subtype of idiopathic generalized epilepsy, its dynamical activities are typically regarded as global brain activities. Accordingly, it is reasonable to assume that the spatial activities during absence seizures are uniform in our model. This implies that the spatial derivative can be ignored in Eq (4). Therefore, the mathematical description of the cortical excitatory axonal field *ϕ*_*e*_ is rewritten as [31, 34–36]:

$$\begin{array}{c} \frac{1}{\gamma_{e}^{2}}\left[\frac{{\text{d}}^{2}}{\text{d}t^{2}}+2\gamma_{e}\frac{\text{d}}{\text{d}t}+\gamma_{e}^{2}\right]\phi_{e}\left(t\right)=Q_{e}\left(t\right). \end{array}$$ (5)

Considering that the intracortical connectivity are proportional to the number of the involved synapses, we can further simplify our model by setting *V*_*i*_ = *V*_*e*_ and *Q*_*i*_ = *Q*_*e*_ for the inhibitory interneurons in the cerebral cortex. Such simplified method has been widely used in previous modelling studies [21, 28–36]. Based on these critical assumptions, the detailed first-order formulations for all neural populations in our modified BGCT model are given in the Supporting Information (see S1 Text).

### Model parameters

Most parameters in our BGCT model are estimated from experimental data and their values are adapted from previous modelling studies (see Table 1) [21, 28–37]. Unless otherwise stated, we use these default parameter values in the following numerical studies. Due to lack of quantitative data, the coupling strength of the direct efferent projection from the GPe to cortical neurons is unknown and requires to be estimated. Because the GPe neurons also send GABAergic projections to STN, GPi and itself, it is reasonable to infer that the coupling strengths of these different pathways projecting from the GPe are comparable. Accordingly, the coupling strength of the direct GABAergic pallido-cortical pathway is considered in the range of 0 to 0.2 mV s in the present work.

**Table 1:** Default parameter values used in this study, which are adapted from previous modelling studies [21, 28–37].

**A: Maximum firing rate**					
**Symbol**	**Value**	**Unit**	**Description**		**References**
$Q_{e}^{max}$, $Q_{i}^{max}$	250	Hz	Cortical maximum firing rate		[21, 33–35]
$Q_{d_{1}}^{max}$, $Q_{d_{2}}^{max}$	65	Hz	Striatum maximum firing rate		[21, 28, 29]
$Q_{p_{1}}^{max}$	250	Hz	SNr/GPi maximum firing rate		[21, 28, 29]
$Q_{p_{2}}^{max}$	300	Hz	GPe maximum firing rate		[21, 28, 29]
$Q_{\zeta }^{max}$	500	Hz	STN maximum firing rate		[21, 28, 29]
$Q_{s}^{max}$	250	Hz	SRN maximum firing rate		[21, 33–35]
$Q_{r}^{max}$	250	Hz	TRN maximum firing rate		[21, 33–35]
**B: Mean firing threshold**					
**Symbol**	**Value**	**Unit**	**Description**		**References**
*θ*_*e*_, *θ*_*i*_	15	mV	Mean firing threshold of cortical populations		[21, 33–35, 37]
*θ*_*d*_1__, *θ*_*d*_2__	19	mV	Mean firing threshold of striatum		[21, 28, 29]
*θ*_*p*_1__	10	mV	Mean firing threshold of SNr/GPi		[21, 28, 29]
*θ*_*p*_2__	9	mV	Mean firing threshold of GPe		[21, 28, 29]
*θ*_*ζ*_	10	mV	Mean firing threshold of STN		[21, 28, 29]
*θ*_*s*_	15	mV	Mean firing threshold of SRN		[21, 33–35, 37]
*θ*_*r*_	15	mV	Mean firing threshold of TRN		[21, 33–35, 37]
**C: Coupling strength**					
**Symbol**	**Value**	**Unit**	**Source**	**Target**	**References**
*v*_*ee*_	1	mV s	Excitatory pyramidal neurons	Excitatory pyramidal neurons	[21, 34, 35]
−*v*_*ei*_	1.8	mV s	Inhibitory interneurons	Excitatory pyramidal neurons	[21, 34, 35]
*v*_*re*_	0.05	mV s	Excitatory pyramidal neurons	TRN	[21, 33, 35]
*v*_*rs*_	0.5	mV s	SRN	TRN	[21, 33, 35]
$-v_{sr}^{A,B}$	0.8	mV s	TRN	SRN	[21, 33, 37]
*v*_*d*_1_*e*_	1	mV s	Excitatory pyramidal neurons	Striatal D1 neurons	[21, 28, 29]
−*v*_*d*_1_*d*_1__	0.2	mV s	Striatal D1 neurons	Striatal D1 neurons	[21, 28, 29]
*v*_*d*_1_*s*_	0.1	mV s	SRN	Striatal D1 neurons	[21, 28, 29]
*v*_*d*_2_*e*_	0.7	mV s	Excitatory pyramidal neurons	Striatal D2 neurons	[21, 28, 29]
−*v*_*d*_2_*d*_2__	0.3	mV s	Striatal D2 neurons	Striatal D2 neurons	[21, 28, 29]
*v*_*d*_2_*s*_	0.05	mV s	SRN	Striatal D2 neurons	[21, 28, 29]
−*v*_*p*_1_*d*_1__	0.1	mV s	Striatal D1 neurons	SNr	[21, 28, 29]
−*v*_*p*_1_*p*_2__	0.03	mV s	GPe	SNr	[21, 28, 29]
*v*_*p*_1_*ζ*_	0.3	mV s	STN	SNr	[21, 28, 29]
−*v*_*p*_2_*d*_2__	0.3	mV s	Striatal D2 neurons	GPe	[21, 28, 29]
−*v*_*p*_2_*p*_2__	0.075	mV s	GPe	GPe	[21, 28, 29]
*v*_*p*_2_*ζ*_	0.45	mV s	STN	GPe	[21, 28, 29]
−*v*_*ζp*_2__	0.04	mV s	GPe	STN	[21, 28, 29]
*v*_*es*_	1.8	mV s	SRN	Excitatory pyramidal neurons	[21, 33, 35]
−*v*_*cp*_2__	0 – 0.2	mV s	GPe	Cerebral cortex	Estimated
*v*_*se*_	2.2	mV s	Excitatory pyramidal neurons	SRN	[21, 33]
*v*_*ζe*_	0.1	mV s	Excitatory pyramidal neurons	STN	[21, 28, 29]
−*v*_*sp*_1__	0.035	mV s	SNr	SRN	[21, 28, 29]
−*v*_*rp*_1__	0.035	mV s	SNr	TRN	[21, 28, 29]
**D: Other parameters**					
**Symbol**	**Value**	**Unit**	**Description**		**References**
*γ*_*e*_	100	Hz	Cortical damping rate		[21, 33–35]
*τ*	50	ms	Time delay due to slow synaptic kinetics of GABA_B_		[21, 30, 35]
*α*	50	s^−1^	Synaptodendritic decay time constant		[21, 33–35, 37]
*β*	200	s^−1^	Synaptodendritic rise time constant		[21, 33–35, 37]
*σ*	6	mV	Threshold variability of firing rate		[21, 33, 34, 37]
*ϕ*_*n*_	2	mV s	Nonspecific subthalamic input onto SRN		[21, 33, 34, 37]

### Analysis of simulation data

We now introduce several data analysis techniques used to quantitatively evaluate the dynamical states generated by our BGCT model. Most of these data analysis techniques are adapted from our previous studies [21, 30], which can be simply summarized as follows.

**Fig 2:**
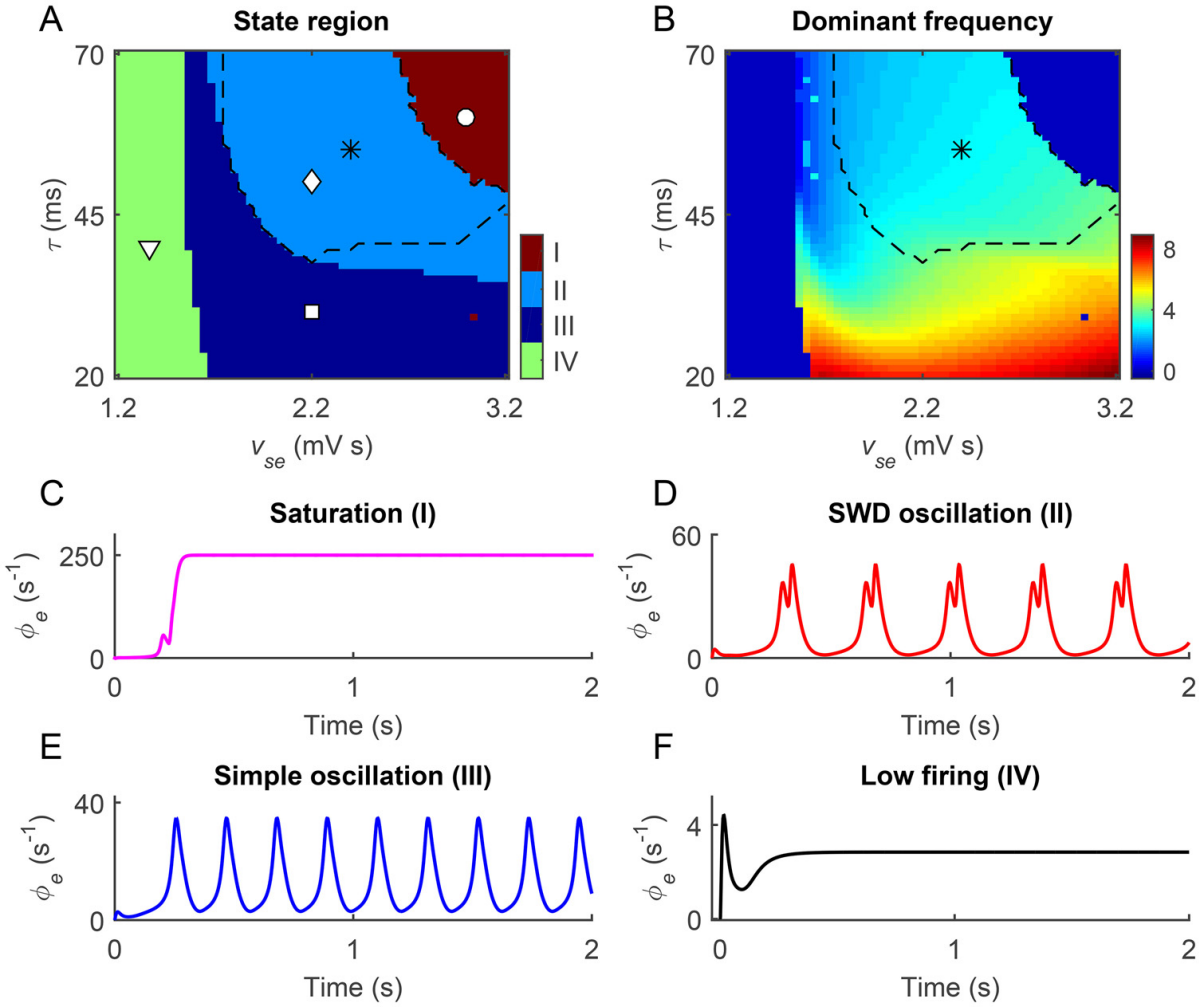
Absence seizures induced by strong coupling of the cortico-thalamic pathway and slow dynamics of GABA_B_ receptors in TRN. A, B: Two-dimensional state analysis (A) and frequency analysis (B) in the (*v_se_*, *τ*) panel. Here *v_se_* represents the excitatory coupling strength of the cortico-thalamic pathway emitting from the pyramidal neurons to SRN, whereas *τ* denotes the GABA_B_ delay. Similar to previous work, four types of dynamical state regions are observed: the saturation region (I), the SWD oscillation region (II), the simple oscillation region (III) and the low firing region (IV). The asterisk (“*”) regions surrounded by black dashed lines in (A) and (B) represent the typical SWD oscillation regions falling into the 2–4 Hz frequency range. C-F: Typical time series of *ϕ*_*e*_ correspond to the above four dynamical states. Four symbols in the state analysis diagram (A) are linked to parameter values used for different typical time series in (C)-(F): I (“∘”), II (“◇”), III (“◻”), and IV (“▿”). Note that we set *v*_*cp*_2__ = −0.05 mV s for all simulations.

First, both the bifurcation and frequency analysis are employed to characterize the critical dynamical transitions and neural oscillations generated by our model [21, 30]. To explore transitions between different dynamical states, the bifurcation analysis is performed for several critical parameters. The bifurcation diagram is obtained by plotting the stable local minimum and maximum values of cortical excitatory axonal fields by gradually changing a critical system parameter. For a specified parameter condition, a series of bifurcation analysis using different independent random initial conditions are performed to detect the possible bistability. For two combined parameters, the above bifurcation analysis allows us to identify different dynamical states in the two-dimensional parameter space (for example, see Fig 2A). To evaluate the dominant frequency of neural oscillations, the power spectral density is estimated using the fast Fourier transform for the time series *ϕ*_*e*_. Then, the maximum peak frequency is defined as the dominant frequency of neural oscillations. By combining both the bifurcation and frequency analysis techniques, the typical 2–4 Hz SWD oscillation region can be roughly outlined in the two-dimensional parameter space (for example, see the asterisk region in Fig 2A).

**Fig 3:**
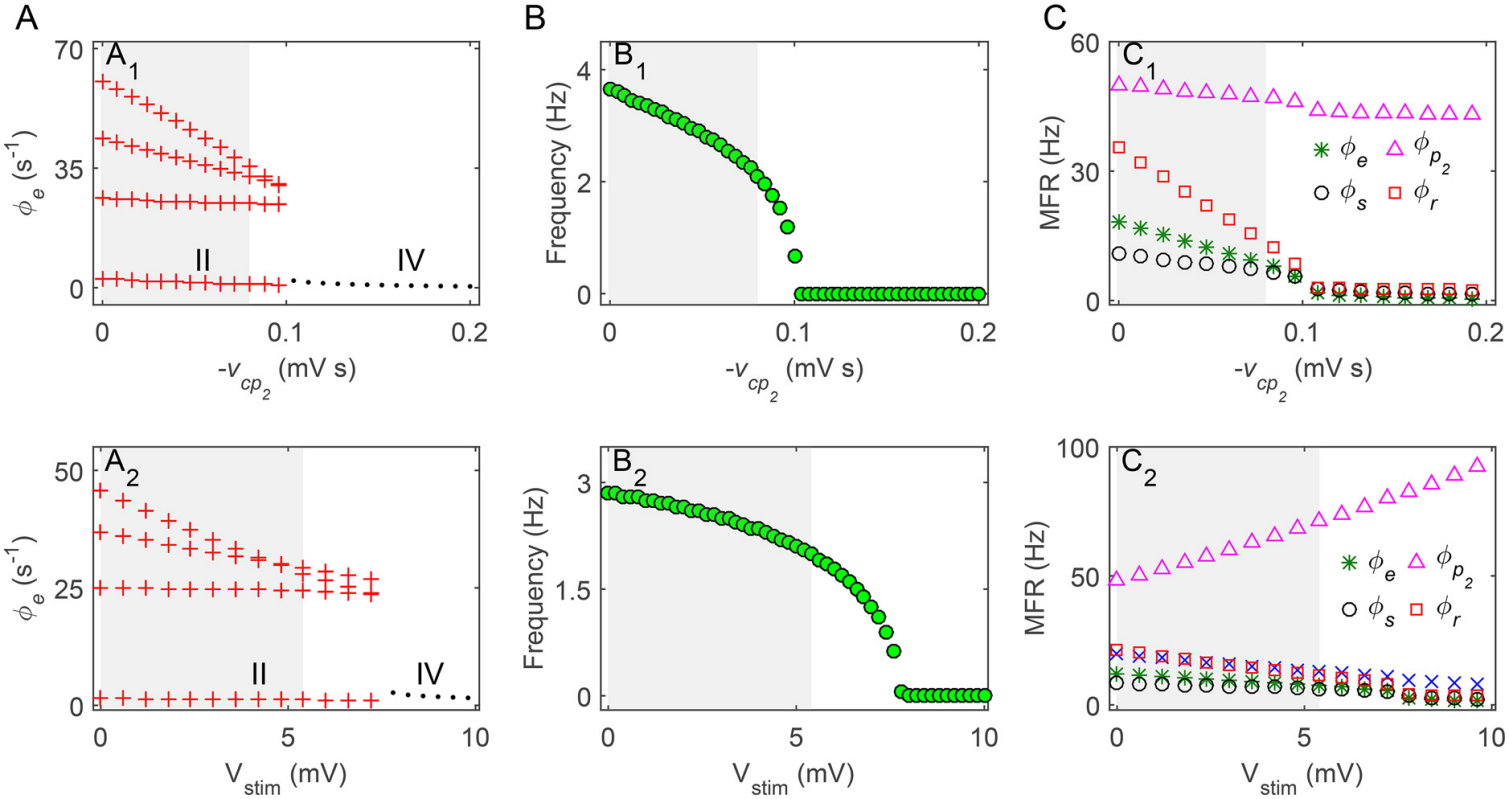
Control of absence seizures by the direct GABAergic pallido-cortical pathway. A: Bifurcation diagrams of *ϕ*_*e*_ as a function of the inhibitory coupling strength of the GABAergic pallidocortical pathway −*v*_*cp*_2__ (A_1_) and the external stimulation *V*_stim_ to GPe neurons (A_2_). It can be seen that both increasing the values of −*v*_*cp*_2__ and *V*_stim_ push the model dynamics from the SWD oscillation region (II) into the low firing region (IV). B: The dominant frequency of neural oscillations as a function of −*v*_*cp*_2__ (B_1_) and *V*_stim_ (B_2_). C: The mean firing rates (MFRs) of several key neural populations as a function of −*v*_*cp*_2__ (C_1_) and V_stim_ (C_2_). Here four neural populations are considered: GPe (“▵”), excitatory pyramidal neurons (“*”), SRN (“∘”) and TRN (“◻”). Note that the gray regions in (A)–(C) denote the SWD oscillations falling into the typical 2–4 Hz.

**Fig 4:**
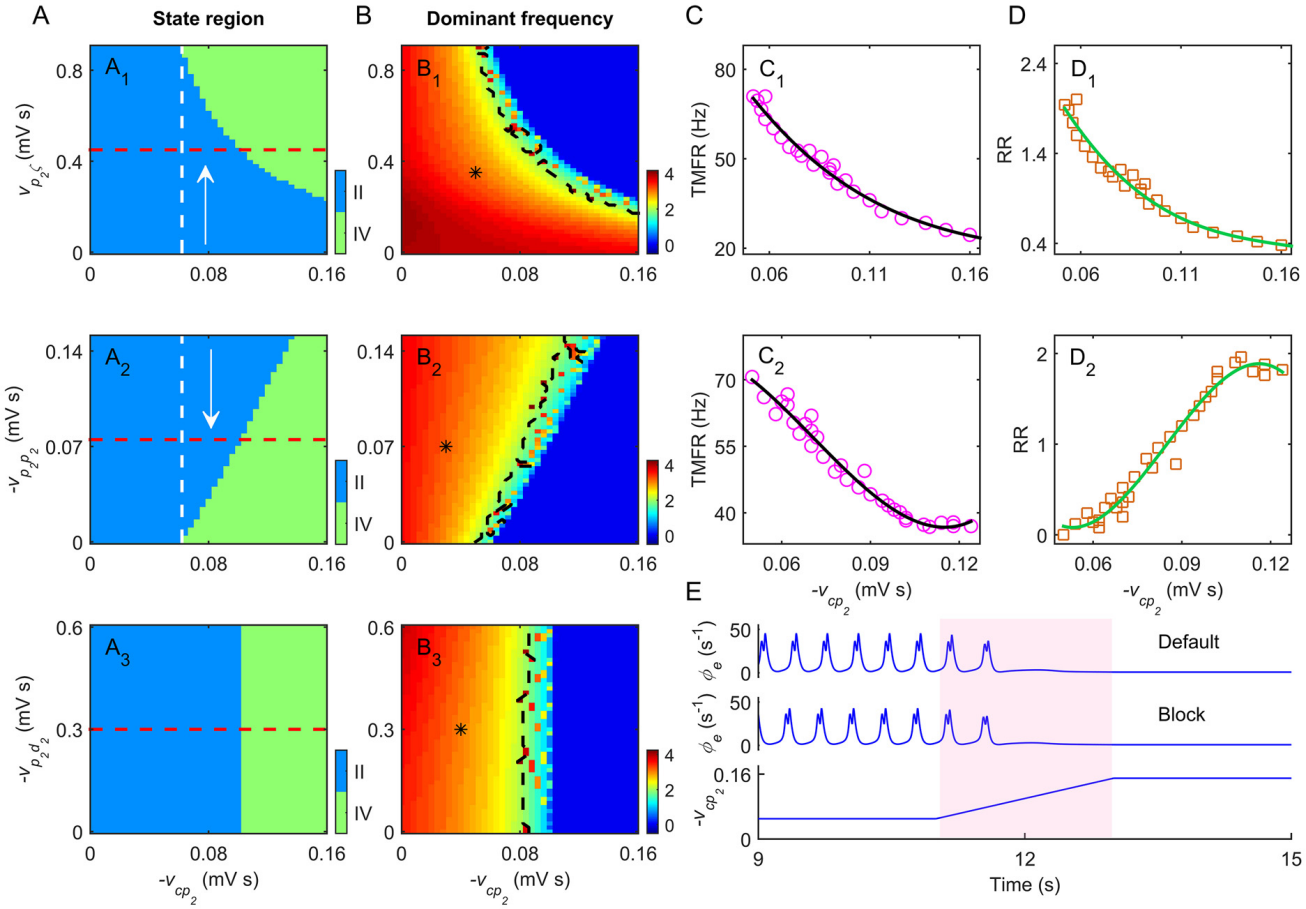
Effects of direct GPe-related pathways on regulating absence seizures. A, B: Two dimensional state analysis (A) and frequency analysis (B) in different parameter spaces. Here we consider three direct GPe-related pathways: the excitatory STN-GPe pathway (A_1_, B_1_), the inhibitory GPe recurrent pathway (A_2_, B_2_) and the inhibitory striatal D2-GPe pathway (A_3_, B_3_), corresponding to parameter spaces (−*v*_*cp*_2__, *v*_*p*_2__ *ζ*), (−*v*_*cp*_2__, −*v*_*p*_2_*p*_2__) and (−*v*_*cp*_2__, −*v*_*p*_2_*d*_2__), respectively. In (A_1_)–(A_3_), two dynamical state regions are observed: the SWD oscillation region (II) and the low firing region (IV). The suppression of SWDs appears to the right of the white dashed line in (A_1_) and (A_2_), where the arrows denote the suppression directions of SWDs. The red lines in (A_1_)-(A_3_) represent the default coupling strengths of these direct GPe-related pathways. The asterisk (“*”) regions surrounded by black dashed lines in (B_1_)-(B_3_) denote the typical 2–4 Hz SWD oscillation regions. C: The triggering mean firing rate (TMFR) as a function of −*v*_*cp*_2__ for the excitatory STN-GPe pathway (C_1_) and inhibitory GPe recurrent pathway (C_2_). D: The relative ratios (RRs) as a function of −*v*_*cp*_2__ for the excitatory STN-GPe pathway (D_1_) and inhibitory GPe recurrent pathway (D_2_). E: Typical time series of *ϕ*_*e*_ by changing −*v*_*cp*_2__ under two conditions of the inhibitory striatal D2-GPe pathway (“default” and “block”). The pink region in (E) denotes the suppression of SWDs by increasing −*v*_*cp*_2__. Obviously, blockade of the inhibitory striatal D2-GPe pathway does not impact the model dynamics significantly.

Furthermore, two measurements related to the firing rate are utilized to explore the underlying mechanism of the SWD suppression [21, 30]. In some figures, we compute the long-term mean firing rates (MFRs) for several critical neural populations. For a given neural population *a*, the long-term MFR is obtained by averaging the value of *Q*_*a*_ in a sufficient long time interval. Such analysis enables us to understand the biophysical mechanism of the SWD suppression caused by the inhibitory pallido-cortical pathway (for example, see Fig 3C). In some cases, we also calculate the critical triggering mean firing rate (TMFR) for the GPe and SNr neurons. Similar to our previous studies [21, 30], the TMFR can be determined by the mean firing rates of a specified type (GPe or SNr) of neurons occurring at the boundaries of the typical region of 2–4 Hz SWDs (for example, see Fig 4B and 4C).

Finally, we also evaluate the relative ratio (RR) of the coupling strength for several important GPe-related pathways. For a given GPe-related pathway, the RR is defined as the critical coupling strength of this GPe-related pathway just triggering the suppression of 2–4 Hz SWDs to its default coupling strength. Theoretically, a RR value close to 1 indicates that the suppression of 2–4 Hz SWDs is easy to accomplish, thus having a relatively high biological plausibility. In several figures, we plot the RR value as a function of the coupling strength of the direct GABAergic pallido-cortical pathway (for example, see Fig 4D). Such a plot allows us to quantitatively estimate the capability of a specified GPe-related pathway in controlling absence seizures in a continuous parameter space.

To obtain convincing results, we carry out 20 trails of simulations for each experimental setting. In the present study, all simulations are performed up to 25 seconds. In each simulation, an initial interval of 5 seconds is included to allow the dynamics of our BGCT model to reach its stable state, and the data from 5 to 25 seconds are used for statistical analysis.

### Simulation method

All numerical simulations are performed using custom codes written in MATLAB (MathWorks, USA). The differential equations are solved by the standard fourth-order Runge-Kutta integration scheme. The temporal resolution of numerical integration is fixed at 0.05 ms. In additional simulations, we have demonstrated that the chosen integration step is small enough to ensure the numerical accuracy of our BGCT model, so that further lowing it does not appreciably affect numerical results. The computer codes used in the present work, combined with the implantation of our previously developed BGCT model, will be available to download from ModelDB (https://senselab.med.yale.edu/ModelDB/showmodel.cshtml?model=152113).

## Results

### Our BGCT model replicates typical absence seizure activities by introducing pathological mechanisms

Past electrophysiological recordings in patients and animals have provided evidence that the abnormal interactions between cerebral cortex and thalamus are responsible for AE [5–8]. Thus, several pathological mechanisms in relation to the corticothalamic system have been proposed to explain the generation of the typical 2–4 Hz SWDs during absence seizures. In particular, recent modelling studies have revealed that both too strong coupling of the cortico-thalamic pathway and excessively slow dynamics of GABA_B_ receptors in TRN play important roles in destroying the normal oscillatory pattern of the corticothalamic system and triggering the onset of absence seizures [33–35, 38].

To investigate whether these pathological mechanisms can induce 2–4 Hz SWDs in our model, we focus on two relevant parameters *v*_*se*_ and *τ*, and perform both bifurcation and frequency analysis in the combined parameter space (see Fig 2A and 2B). Similar to previous results [21, 30], four types of dynamical states appear in different parameter regions (Fig 2A). When the excitatory coupling *v*_*se*_ is too strong and the GABA_B_ delay *τ* is too long, the inhibition from the TRN has rather week effect on SRN neurons. Under this condition, the recurrent excitation between the cerebral cortex and SRN drives the firing of cortical pyramidal neurons to their saturation states in a short time (region I in Fig 2A and Fig 2C). For appropriate combination of *v*_*se*_ and *τ*, the suppression of SRN due to independent GABA_A_- and GABA_B_-mediated TRN-SRN pathways is effective and occurs at different time instants. Such double suppression effect shapes the firing of SRN, which further influences the dynamics of cortical neurons, leading to the generation of SWD oscillation state in which multiple pairs of maximum and minimum values are found within each periodic complex (region II in Fig 2A and Fig 2D). Interestingly, we find that most of this region falls into the typical 2–4 Hz frequency range (asterisk regions in Fig 2A and 2B), which is of importance because 2–4 Hz SWDs have been widely observed on the EEG recordings of real AE patients [5]. Note that, for an intermediate *v*_*se*_, our model requires a minimal level of GABA_B_ delay to ensure the occurrence of SWDs [21]. Theoretically, if the interval between the GABA_A_- and GABA_B_-induced inhibitions is too close, these two signals will fuse well together and the double suppression effect is considerably weakened. In this case, neural oscillations of cortical neurons are deteriorated to the simple oscillation state (region III in Fig 2A and Fig 2E). When the excitatory coupling *v*_*se*_ is too weak, the firing of SRN is almost completely inhibited by TRN neurons. Thus, the dynamics of our model is pushed into the low firing state and no oscillation is observed anymore (region IV in Fig 2A and Fig 2F).

These results showed that the modified BGCT model with the new identified GABA_A_-mediated pallido-cortical pathway can mimic different types of dynamical states of the brain. In particular, we confirmed that our model can successfully replicate typical absence seizure activities by introducing suitable pathological mechanisms. In the following studies, we set *v*_*se*_ = 2.2 mV s and *τ* = 50 ms by default, and explore whether and how the BG regulate typical absence seizure activities through the inhibitory pallido-cortical pathway.

### Controlling absence seizures through the direct GABAergic pallido-cortical pathway

Recent experimental data identified the existence of direct GABAergic pallido-cortical pathway projecting from GPe neurons to both excitatory and inhibitory neurons in the cerebral cortex [22, 23]. Theoretically, outputs from this inhibitory pathway are able to shape the firing of cortical neurons, which might provide a potential mechanism to regulate absence seizures. To examine whether our hypothesis is correct, two relevant experimental approaches are designed. In the first approach, we focus on the inhibitory coupling strength of the pallido-cortical pathway −*v*_*cp*_2__ and explore its effect on controlling absence seizures. In the second approach, we keep the value of −*v*_*cp*_2__ as a constant and introduce a positive external stimulation *V*_stim_ to GPe neurons. Using this method, we can modulate the firing rate of GPe neurons in a highly controlled manner and investigate whether the activation level of GPe neurons also participates in the control of absence seizures.

Fig 3A illustrates the bifurcation diagrams for parameters −*v*_*cp*_2__ and *V*_stim_. As expected, we observe that both increasing the coupling strength of the inhibitory pallido-cortical pathway and enhancing the intensity of the external stimulation to GPe neurons push the model dynamics from the SWD oscillation state into the low firing state. Unlike the typical SWD suppression presented in our previous studies [21, 30], the transition between these two types of dynamical states is abrupt and does not undergo the simple oscillation state, indicating that the pallido-cortical pathway induced SWD suppression is somewhat strong. Further investigation using frequency analysis shows that the dominant frequency of SWDs oscillation is also influenced by −*v*_*cp*_2__ and *V*_stim_ (Fig 3B). The increase in their strengths gradually reduces the dominant frequency of neural oscillations. As a consequence, even in the SWD oscillation region, the dominant frequency of SWDs is pushed below the typical frequency range of 2–4 Hz (gray regions in Fig 3) for sufficiently strong −*v*_*cp*_2__ and *V*_stim_. These results suggest that the BG can effectively terminate typical absence seizure activities by the strong inhibitory effect from the GABA_A_-mediated pallido-cortical pathway.

To mechanistically understand how the pallido-cortical pathway induced SWD suppression arises, we compute the long-term mean firing rates of several key neural populations within the BGCT system for these two designed approaches. As shown in Fig 3C, we find that increasing the coupling strength −*v*_*cp*_2__ slightly reduces the firing rate of GPe neurons, whereas enhancing the external stimulation *V*_stim_ solely improves the firing rate of GPe neurons. In these two cases, the average effective influences from GPe neurons to cortical neurons (i.e., −*v*_*cp*_2__ *F*[*V*_*p*_2__]) are overall enhanced with the growth of −*v*_*cp*_2__ and *V*_stim_, thus leading to the firing reduction for cortical neurons. The inactivation of cortical neurons tends to decrease the firing rate of SRN neurons, which in turn reduces the activation of cortical neurons through the local feedback excitation loop. Together, these chain reactions result in a pronounced inhibition of cortical neurons, further reducing the firing of TRN neurons. For sufficiently strong −*v*_*cp*_2__ and *V*_stim_, the inactivation of TRN greatly weakens the double peak shaping effect due to the slow kinetics of GABA_B_ receptors in TRN. This GABA_B_ weakening may thus provide an effective biophysical mechanism to cause the suppression of SWDs.

Our above findings provide the first computational evidence that the BG may control and modulate absence seizures through the direct GABAergic pallido-cortical pathway. To be specific, we demonstrate that both increasing the coupling strength of the inhibitory pallido-cortical pathway and enhancing the activation of GPe neurons could significantly suppress the generation of typical 2–4 Hz SWDs. It is reasonable to further speculate that several GPe-related pathways, which directly and indirectly regulate the activation level of GPe neurons, may also trigger the SWD suppression through the inhibitory pallido-cortical pathway.

### Several direct GPe-related pathways play active roles in controlling absence seizures

Here we explore the effects of the input and recurrent pathways of GPe on controlling typical absence seizure activities. As shown in Fig 1, the GPe receives excitatory and inhibitory projections from STN and striatal D2 neurons, and also it has recurrent inhibitory projection from itself. Outputs from these direct GPe-related pathways modulate the firing of GPe neurons directly, thus might play critical roles in regulating absence seizures.

We first focus on the excitatory STN-GPe pathway and the inhibitory GPe recurrent pathway, and estimate their contributions to the control of absence seizures. To this end, two groups of state and frequency analysis are performed in the combined (−*v*_*cp*_2__,*v*_*p*_2_*ζ*_) and (−*v*_*cp*_2__, −*v*_*p*_2_*p*_2__) parameter spaces (see Fig 4A and 4B). Our results suggest that both the suppression of SWDs and the dominant frequency of neural oscillations are modulated by these two pathways. Theoretically, the increase in the excitatory coupling strength of the STN-GPe pathway promotes the activation level of GPe neurons, whereas the increase in the inhibitory coupling strength of the GPe recurrent pathway inactivates the GPe neurons. In agreement with our above results, we observe that both increasing the excitatory coupling strength *v*_*p*_2_*ζ*_ and decreasing the inhibitory coupling strength −*v*_*p*_2_*p*_2__ reduce the dominant frequency of SWDs, and lead to SWD suppression in strong −*v*_*cp*_2__ region. However, such SWD suppression greatly relies on the strength of the GABAergic pallido-cortical pathway. For a strong −*v*_*cp*_2__, outputs from the GPe neurons inhibit the firing of cortical neurons significantly. In this case, our BGCT model works in the low firing state for default values of *v*_*p*_2_*ζ*_ and −*v*_*p*_2_*p*_2__ (Fig 4A), and the SWD suppression occurs at relatively weak *v*_*p*_2_*ζ*_ and strong −*v*_*p*_2_*p*_2__ (compared to their default values).

Because the activation of GPe neurons is improved by both increasing *v*_*p*_2_*ζ*_ and decreasing −*v*_*p*_2_*p*_2__, our above results also indicate that the developed model might have the corresponding critical triggering mean firing rates (TMFR) for GPe neurons (Fig 4A, single arrow). If the long-term mean firing rate of GPe neurons lower than this TMFR, our model can highly generate typical 2–4 Hz SWDs. In Fig 4C_1_ and 4C_2_, we plot the value of TMFR as a function of −*v*_*cp*_2__ for the STN-GPe pathway and the GPe recurrent pathway, respectively. For both cases, it is observed that this critical TMFR reduces basically with increasing the strength of the inhibitory pallido-cortical pathway. This is not so surprising because, during the growth of −*v*_*cp*_2__, the average effective influence from GPe neurons to cortical neurons still maintains a relatively high level, which is sufficiently strong to suppress the generation of 2–4 Hz SWDs. Note that the similar but more complicated TMFRs are also discovered for the SNr neurons in our previous work [21]. In that study, two types of TMFRs (i.e., the low and high TMFRs) are identified for SNr neurons, and both activating and inactivating the SNr neurons from the normal level may effectively terminate the generation of SWDs [21].

To quantitatively estimate the underlying suppression capabilities of the excitatory STN-GPe pathway and the inhibitory GPe recurrent pathway, we further calculate the relative ratio of coupling strength as a function of −*v*_*cp*_2__ for these two direct GPe-related pathways (see Fig 4D). For each pathway, it is found that only a slight tuning of the corresponding coupling strength from its default value is required to terminate the typical 2–4 Hz SWDs (Fig 4D_1_ and 4D_2_). This suggests that appropriately changing each of these two direct GPe-related pathways results in a pronounced firing enhancement in GPe neurons, which further strongly regulates absence seizures. To a certain extent, our above results obtained by quantitative evaluation indicate that the SWD suppression triggered by these two pathways has relatively high biological plausibility.

We then turn to the inhibitory striatal D2-GPe pathway and investigate whether this pathway also takes part in controlling absence seizures. In Fig 4A_3_ and 4B_3_, we show the two-dimensional state and frequency analysis in the (−*v*_*cp*_2__, −*v*_*p*_2_*d*_2__) parameter space, respectively. In contrast with our above results, we find that decreasing the inhibitory coupling strength −*v*_*p*_2_*d*_2__ almost does not influence the dynamical states generated by our model. For insufficiently strong −*v*_*cp*_2__, the failure of the SWD suppression can be observed even when the striatal D2-GPe pathway is completely blocked (i.e., *v*_*p*_2_*d*_2__ = 0 mV s, and see Fig 4E). Under this condition, the striatal D2 neurons do not inhibit the GPe neurons at all, so that the underlying modulation capability of the striatal D2-GPe pathway is theoretically amplified to the extreme. This finding suggests that inactivating striatal D2 neurons contributes limited to the firing enhancement in GPe neurons, thus failing to assist the triggering of the SWD suppression purely through the inhibitory pallido-cortical pathway.

Our above computational data indicate that both the excitatory STN-GPe pathway and the inhibitory GPe recurrent pathway participate in controlling absence seizures as well. Appropriate tuning of these two direct GPe-related pathways may also trigger the SWD suppression by strongly modulating the activation level of GPe neurons. Therefore, we believe that the contributions of these two direct GPe-related pathways to absence seizure control are eventually accomplished through the GABAergic pallido-cortical pathway.

### Indirect GPe-related pathways have relatively weak effects on controlling absence seizures

In addition to direct GPe-related pathways, the activation level of GPe neurons might be also mediated by the firing activities from several indirect GPe-related pathways. Intuitively, there are two important underlying pathways in our model. The first one is the inhibitory GPe-STN pathway and the other one is the excitatory hyperdirect pathway from cortical pyramidal neurons to STN. Theoretically, outputs from these two pathways modulate the firing of STN in an direct manner, and then further influence the activation level of GPe neurons.

**Fig 5:**
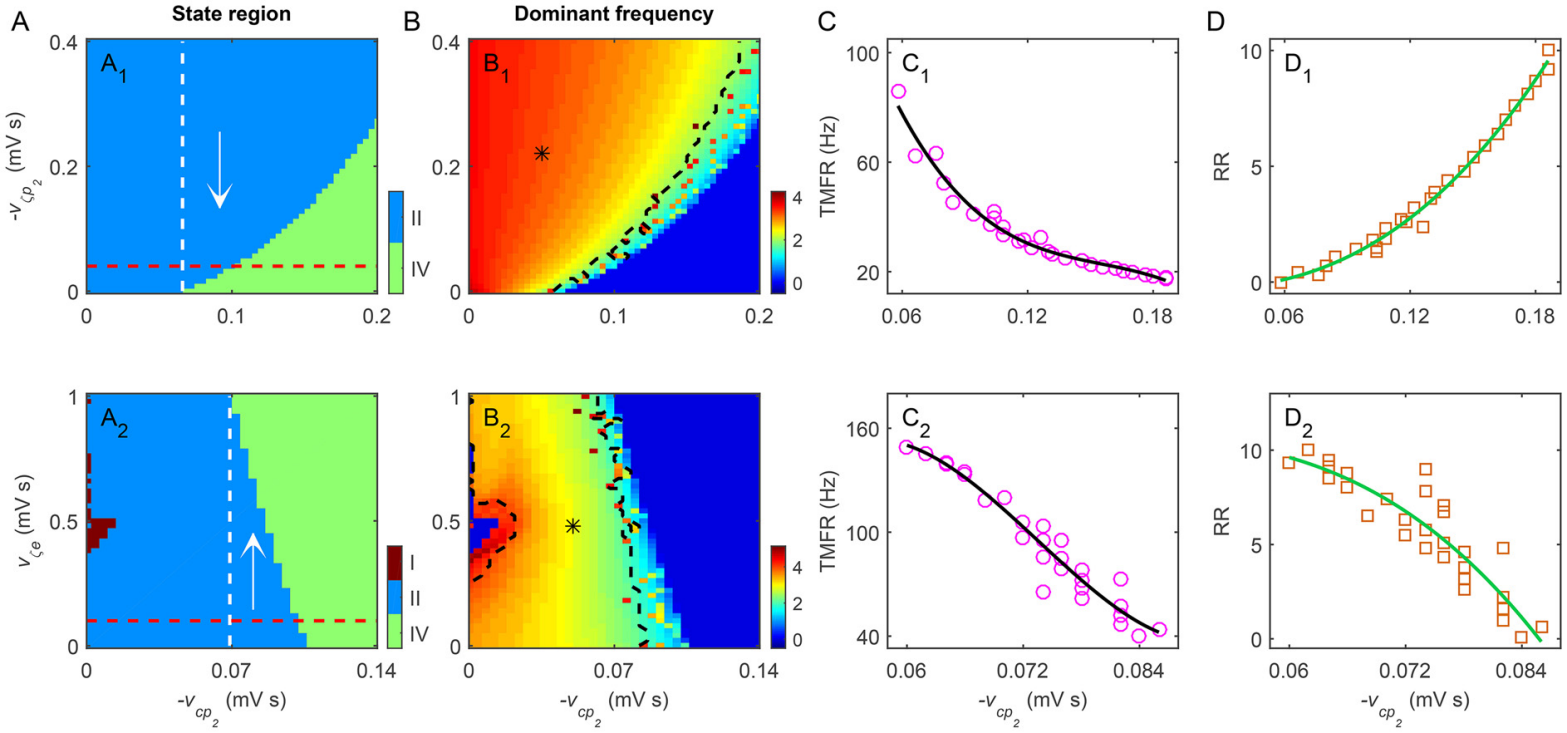
Effects of indirect GPe-related pathways on regulating absence seizures. A, B: Two-dimensional state analysis (A) and frequency analysis (B) in the combined (−*v*_*cp*_2__, −*v*_*ζp*_2__) and (−*v*_*cp*_2__, −*v*_*ζe*_) parameter spaces. Two considered indirect GPe-related pathways are: the inhibitory GPe-STN pathway (A_1_, B_1_) and the excitatory hyperdirect pathway from pyramidal neurons to STN (A_2_, B_2_). Three dynamical state regions are observed in the state analysis diagrams: the saturation region (I), the SWD oscillation region (II) and the low firing region (IV). In (A_1_) and (A_2_), the red dashed lines stand for the default coupling strengths of these two indirect GPe-related pathways, the white dashed lines represent the boundaries of suppression regions of SWDs, and the arrows denote the suppression directions of SWDs. In (B_1_) and (B_2_), the asterisk (“*”) regions surrounded by black dashed lines are the SWD oscillation regions falling into the 2–4 Hz frequency range. C: The TMFR as a function of −*v*_*cp*_2__ for the inhibitory GPe-STN pathway (C_1_) and the excitatory hyperdirect pathway (C_2_). D: The RR as a function of −*v*_*cp*_2__ for the inhibitory GPe-STN pathway (D_1_) and the excitatory hyperdirect pathway (D_2_). Compared to the results in Fig 4, these two indirect GPe-related pathways have relatively weak effects on controlling absence seizures.

To determine whether these indirect GPe-related pathways also contribute to the control of absence seizures, we implement both the two-dimensional state and frequency analysis in the combined (−*v*_*cp*_2__, −*v*_*ζp*_2__) and (−*v*_*cp*_2__,*v*_*ζe*_) parameter spaces (see Fig 5A and 5B). As expected, the decrease in the inhibitory coupling strength of the GPe-STN pathway causes the firing enchantment of STN, which in turn improves the activation of GPe neurons. In accordance with our above findings, this leads to the suppression of typical 2–4 Hz SWDs in strong −*v*_*cp*_2__ region (Fig 5A_1_ and 5B_1_). On the other hand, the increase in the excitatory coupling strength of the hyperdirect pathway activates both the STN and GPe neurons, thus also resulting in the suppression of typical 2–4 Hz SWDs in strong −*v*_*cp*_2__ region (Fig 5A_2_ and 5B_2_). Similar to our above results, we find that the model also exhibits the critical TMFR for GPe neurons in the SWD suppression region for each pathway. For a given −*v*_*cp*_2__ within the suppression region, the generation of 2–4 Hz SWDs can be highly triggered provided that the long-term mean firing rate of GPe neurons is lower than this critical TMFR. With the increasing of −*v*_*cp*_2__, this critical TMFR reduces rapidly for both the inhibitory GPe-STN pathway and excitatory hyperdirect pathway. However, compared to the results in Fig 4D, our results show that the suppression of typical absence seizure activities generally occurs at quite strong −*v*_*ζp*_2__ and *v*_*ζe*_ regions (several folds of their default values, see Fig 5D). To a certain degree, this finding might imply that the 2–4 Hz SWD suppression caused by these two indirect GPe-related pathways might be biophysical difficulty to realize, and thus has relatively low biological plausibility.

By combining all results in these two subsections, we conclude that both the inhibitory GPe-STN pathway and excitatory hyperdirect pathway contribute to the regulation of typical 2–4 Hz SWDs, but they might play relatively weak roles in controlling absence seizures compared to those two direct GPe-related pathways identified above. For comparison, the effects of both direct and indirect GPe-related pathways on absence seizure control are simply summarized in Table 2.

**Table 2:** Roles of several GPe-related pathways in the regulation of absence seizures, through modulating the activation level of GPe neurons.

**Source**	**Target**	**Type**	**Control manner**	**Biological plausibility**
STN	GPe	Direct	Increase in *v*_*p*_2_*ζ*_	High
GPe	GPe	Direct	Decrease in −*v*_*p*_2_*p*_2__	High
Striatal D2 neurons	GPe	Direct	No significant effect	Low
GPe	STN	Indirect	Decrease in −*v*_*ζp*_2__	Low
Excitatory pyramidal neurons	STN	Indirect	Increase in *v*_*ζe*_	Low

### The GABAergic pallido-cortical pathway modulates the bidirectional control of absence seizures caused by direct inhibitory nigro-thalamic pathways

Using a BGCT model developed previously, we have shown that the BG may control absence seizures in a bidirectional manner induced by the competition between the SNr-TRN and SNr-SRN pathways [21, 30]. A natural question to ask is: can we observe the similar bidirectional control of absence seizures in our current BGCT model by incorporating the direct GABAergic pallido-cortical pathway? To answer this question, we introduce a scale factor *K* = *v*_*rp*_1__/*v*_*sp*_1__, representing the relative strength between the SNr-TRN and the SNr-SRN pathways. Similar to our previous studies [21, 30], the coupling strength of the excitatory STN-SNr pathway (i.e., *v*_*p*_1_*ζ*_) is employed to control the activation level of SNr neurons. With this method, it is possible to determine whether the competition-induced bidirectional control of absence seizures also exists in our current model.

**Fig 6:**
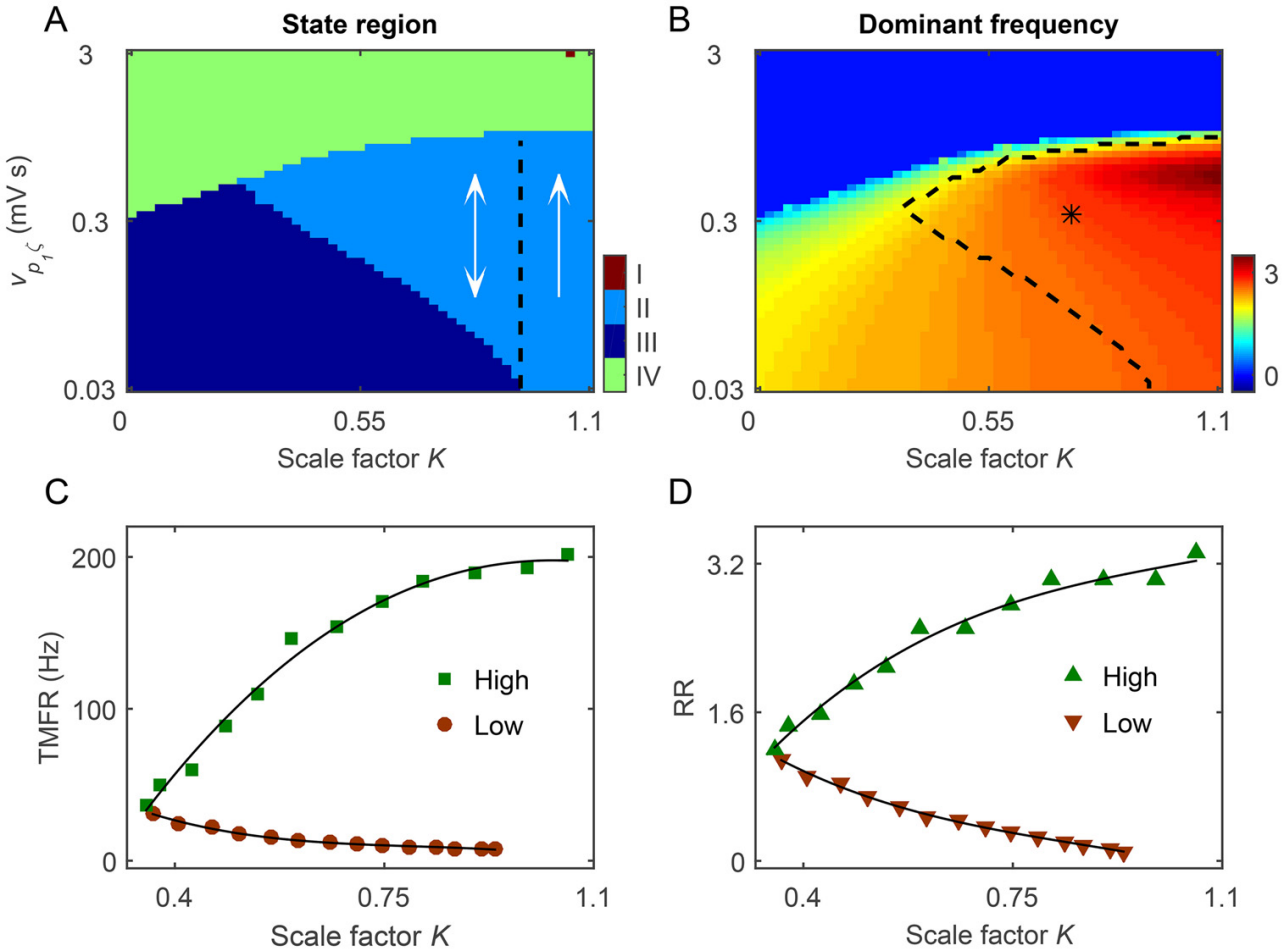
Bidirectional control of absence seizures due to the competition between the SNr-TRN and SNr-SRN pathways. A, B: The state analysis (A) and frequency analysis (B) in the (*K*, *v*_*p*_1_*ζ*_) panel. Here *K* is the scale factor, and *v*_*p*_1_*ζ*_ is the excitatory coupling strength of the STN-SNr pathway. The BGCT model mainly exhibits three types of dynamical states: the SWD oscillation region (II), the simple oscillation region (III) and the low firing region (IV), but occasionally displays the saturation state in the large *K* and strong *v*_*p*_1_*ζ*_ region. For intermediate scale factor *K*, both increase and decrease in the activation level of SNr can inhibit the SWDs (double arrow, bidirectional suppression). In (A), the black dashed line represents the demarcation between the bidirectional (double arrow) and unidirectional suppression (single arrow) regions. The asterisk (“*”) region surrounded by dashed lines in (B) denotes the SWD oscillation region that falls into the 2–4 Hz frequency range. C: The low and high TMFRs of SNr neurons as a function of *K*. D: The low and high RRs of the STN-SNr pathway as a function of *K*. In all simulations, we set *τ* = 45 ms and *v*_*cp*_2__ = −0.06 mV s.

The results of Fig 6A and 6B depict the state and frequency analysis in the (*K*,*v*_*p*_1_*ζ*_) panel. As we can see in Fig 6A, our current BGCT model mainly exhibits three types of dynamical states: the SWD oscillation region (II), the simple oscillation region (III) and the low firing region (IV), but occasionally displays the saturation state in the large *K* and strong *v*_*p*_1_*ζ*_ region. Due to the competition between the SNr-TRN and SNr-SRN pathways, a typical bidirectional control feature is observed for intermediate scale factor *K*. In this bidirectional region, we find that both enhancing and lowing the excitatory coupling strength *v*_*p*_1_*ζ*_ inhibit the generation of 2–4 Hz SWDs, but in different manners. Specifically, a significant improvement in *v*_*p*_1_*ζ*_ kicks the model dynamics into the low firing state, whereas a pronounced reduction in *v*_*p*_1_*ζ*_ pushes the model dynamics into the simple oscillation state (Fig 6A). Because the activation level of SNr neurons is increased with the growth of the excitatory coupling strength *v*_*p*_1_*ζ*_, our above observations thus suggest that the current BGCT model has both the low and high TMFRs for the SNr neurons in this bidirectional region. For a suitable scale factor *K*, the generation of SWDs within the typical 2–4 Hz can be highly triggered provided that the mean firing rate of SNr neurons is between these two critical TMFRs. Such two critical TMFRs are modulated by the relative strength between the SNr-TRN and the SNr-SRN pathways. As the growth of the scale factor *K*, the high TMFR is increased from a low value to saturation, whereas the low TMFR is reduced from a relatively high value to 0 (Fig 6C). Unsurprisingly, we find that the model also has both the low and high RRs for the STN-SNr pathway (Fig 6D). Indeed, these two RRs correspond to above identified two types of TMFRs, exhibiting the similar modulation trends as those observed for the low and high TMFRs (compared the results in Fig 6C and 6D).

**Fig 7:**
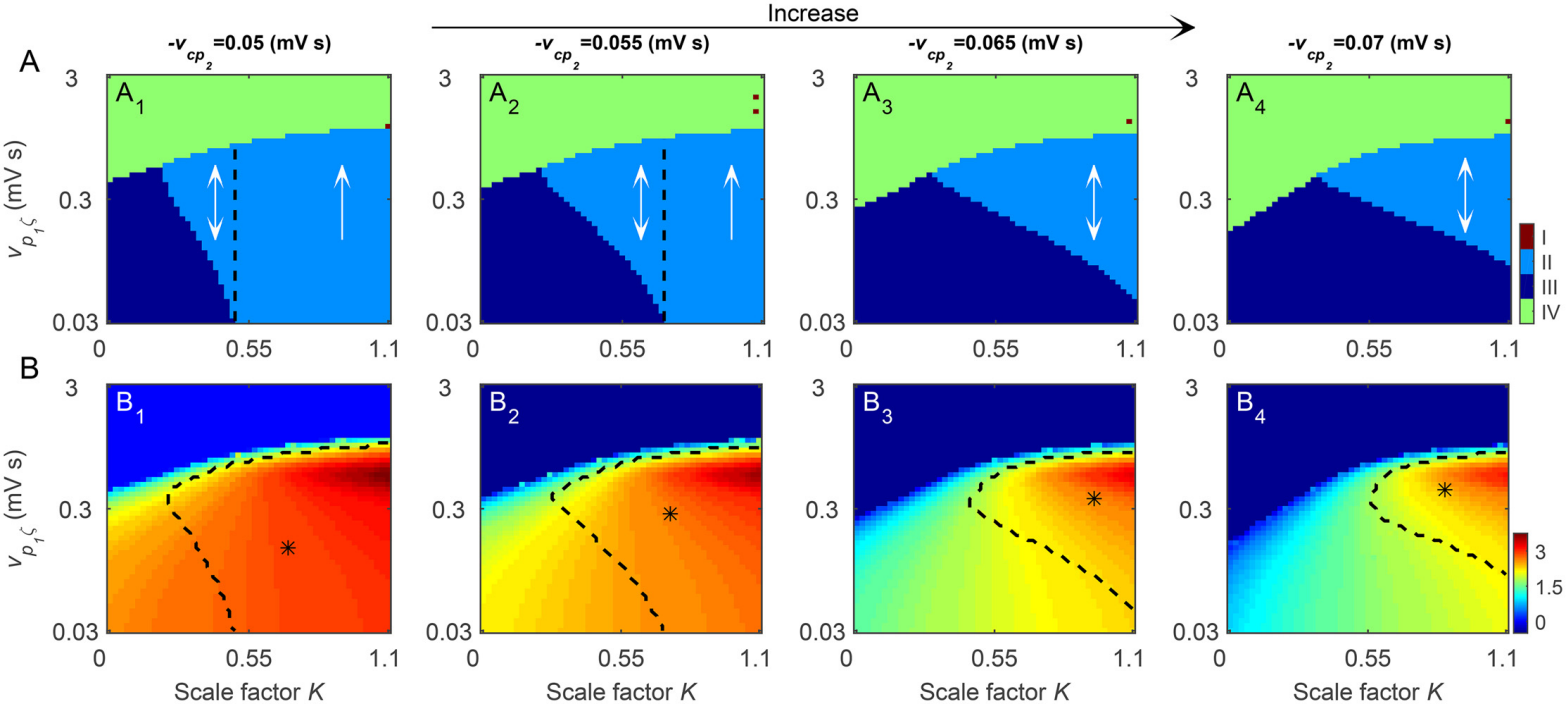
Shaping effects of the direct GABAergic pallido-cortical pathway on the bidirectional control of absence seizures by the BG. A, B: Tow-dimensional state analysis (A) and frequency analysis (B) in the (*K*, *v*_*p*_1_*ζ*_) panel for different values of *v*_*cp*_2__. Similar to the results in Fig 6A, our BGCT model mainly exhibits three types of dynamical states: the SWD oscillation region (II), the simple oscillation region (III) and the low firing region (IV), but occasionally displays the saturation state in the large *K* and strong *v*_*p*_1_*ζ*_ region. In (A_1_)-(A_4_), the double arrows denote the bidirectional suppression and the single arrows represent the unidirectional suppression. The black dashed lines in (A_1_) and (A_2_) stand for the demarcations between the bidirectional and unidirectional suppression regions. In (B_1_)-(B_4_), the asterisk (“*”) regions surrounded by dashed lines denote the regions of 2–4 Hz SWDs. From left to right, the strengths of direct GABAergic pallido-cortical pathway are: *v*_*cp*_2__ = −0.05 mV s (A_1_, B_1_), *v*_*cp*_2__ = −0.055 mV s (A_2_, B_2_), *v*_*cp*_2__ = −0.065 mV s (A_3_, B_3_), and *v*_*cp*_2__ = −0.07 mV s (A_4_, B_4_), respectively. In all simulations, we set *τ* = 45 ms.

More interestingly, we observe that both the suppression of SWDs and the typical 2–4 Hz SWD region are shaped by the strength of the direct GABAergic pallido-cortical pathway. In Fig 7A and 7B, we plot a series of two-dimensional state and frequency analysis in the (*K*,*v*_*p*_1_*ζ*_) panel for different values of −*v*_*cp*_2__. From the theoretical perspective, the increase in the inhibitory coupling strength −*v*_*cp*_2__ reduces the firing of both the TRN and SRN neurons, but contributes more to SRN neurons due to the local feedback excitation loop between pyramidal neurons and SRN (see Fig 3C_1_). To a certain degree, such imbalance inhibition to these two critical thalamic nuclei enlarges the relative effect of the inhibitory SNr-TRN pathway. It is obvious that the higher the coupling strength −*v*_*cp*_2__, the stronger the relative inhibition effect caused by the SNr-TRN pathway. With the increasing of −*v*_*cp*_2__, such strengthened inhibition drives the low TMFR toward higher values of *v*_*p*_1_*ζ*_ (Fig 7A) and therefore shrinks the region of SWDs within the typical frequency range of 2–4 Hz (Fig 7B). Notably, we have also observed that the inhibitory strength of the TRN-SRN pathways has the similar shaping effect on bidirectional control of absence seizures in our previous work [21].

These results confirm that the competition-induced bidirectional control of absence seizures also exists in our current BGCT model and is modulated by the strength of the direct GABAergic pallido-cortical pathway. Taking all recent computational evidence together [21, 30], we postulate that such bidirectional control caused by the BG is possible a generalized regulatory mechanism for absence seizures. However, it should be noted that the competition between the SNr-TRN and SNr-SRN pathways has a determinative role in the bidirectional control of absence seizures, and the typical bidirectional control feature only appears at appropriate levels of competition.

## Discussion

By adapting our previously developed mean-field BGCT model [21, 30], we have investigated the underlying roles of the novel identified GABAergic projection from the GPe to the cerebral cortex in controlling absence seizures in the present study. We demonstrated that both increasing the activation of GPe neurons from the normal level and enhancing the coupling strength of this direct inhibitory pallido-cortical pathway significantly suppress typical 2–4 Hz SWDs during absence seizures. Mechanistically, such SWD suppression is due to the GABA_B_ weakening caused by strong inhibitory outputs from the pallido-cortical pathway. Moreover, we evaluated the effects of different GPe-related pathways on controlling absence seizures. Our results showed that several important GPe-related pathways, which directly and indirectly regulate the activation level of GPe neurons, also contribute to the regulation of typical absence seizure activities. Specifically, both the excitatory STN-GPe pathway and the inhibitory GPe recurrent pathway were identified to play active roles in controlling absence seizures, whereas several indirect GPe-related pathways have relatively weak effects on the control of absence seizures. These findings provide the first theoretical evidence that the BG may regulate absence seizures through the direct GABAergic pallido-cortical pathway. Past experimental and computational studies have shown that applying suitable deep brain stimulations (DBSs) to several critical nuclei in basal ganglia, such as the STN and SNr, can inhibit the production of 2–4 Hz SWDs during absence seizures [39–41]. Our results might suggest that the GPe is possible an alternative DBS therapeutic target for treating absence seizures.

In our previous studies, we have demonstrated the BG could control absence seizures in a bidirectional manner through the direct SNr-TRN and SNr-SRN pathways [21, 30]. Due to the competition between these two inhibitory nigro-thalamic pathways, both increasing and decreasing the activation of SNr neurons from the normal level could considerably suppress the occurrence of SWDs. Here we confirmed that the similar bidirectional control of absence seizures also exists in our modified BGCT model. To a certain extent, this finding suggests that such bidirectional control caused by these two inhibitory nigro-thalamic pathways may be a generalized regulatory mechanism for absence seizures. Taken together, our results presented here and previously emphasize the functional roles of several direct inhibitory pathways, emitting from important BG nuclei to the cerebral cortex and thalamus, in controlling and modulating absence seizures [21, 30].

Moreover, we showed that inactivation of striatal D2 neurons might not improve the firing of GPe neurons in a significant manner, thus failing to assist the triggering of the SWD suppression purely through the inhibitory pallido-cortical pathway. Past experiments in genetic absence epilepsy rats, however, have demonstrated that activation of D2 receptors by intrastriatal injections of D2 agonists, resulting in cell inhibition mediated by a decrease in adenylate cyclase activity through Gi proteins, can considerably suppress absence seizures [17]. Such antiepileptic effect was supposed to be attributed to the inhibition of SNr neurons by the indirect striato-nigral pathway relaying at GPe, which might further trigger the suppression of SWDs via both the direct and indirect nigro-thalamic pathways. Essentially, our computational results are not collided with previous experimental findings, even though the SWD suppression was not observed by inactivating striatal D2 neurons. In this study, such failure might be caused by the lack of indirect nigro-thalamic pathway relaying at superior colliculus in our BGCT model, which is due to the lack of quantitative anatomical data and can be seen as a limitation of our model. Instead, our computational results combined with the previous experimental findings in [17] might inspire that activating GPe neurons have complicated effects on controlling absence seizures through multiple pathways emitting from the BG to the cerebral cortex and thalamus. In the brain of absence epileptic patients, these multiple pathways might work together and play complementary roles, thus providing a stable mechanism to terminate the onset of absence epilepsy.

In addition to the corticothalamic system, some persons might ask whether the basal ganglia also participate in the initiation of absence seizures. Theoretically, the BG have a central position in the brain and involve several important nuclei [15, 16], which may allow them to play crucial roles in the generation of typical absence seizure activities. However, we argue that this might be not true, because the generation of 2–4 Hz SWDs in our model is mainly caused by other two pathogenic factors: too strong coupling of the cortico-thalamic pathway and excessively slow dynamics of GABA_B_ receptors in TRN. If these two pathogenic factors are removed, our model cannot reproduce typical absence seizure activities anymore. Taking all these evidence together, we feel that abnormal alterations from the BG to both the cerebral cortex and thalamus might assist the generation of absence seizures, but these abnormal interactions are not the major pathogenic factors of absence epilepsy. Remarkably, evidence from previous experimental data also failed to support a generator role for the basal ganglia in the initiation of absence seizure activities [42–44], which is consistent with our current viewpoint.

Our modified BGCT model is originally developed for studying absence seizures, but it is extendable to investigate other mental illness and high-level brain functions related to GPe. For instance, the Parkinson’s disease is a brain disorder in relation to motor control, and the BG are believed to be centrally implicated in parkinsonism. Experimental data have suggested that the most important feature of parkinsonism is enhanced oscillations in the population neuronal activity in the beta band (13–30 Hz) [45–47]. Recent modelling studies further implicated that such pronounced beta oscillations in the parkinsonian state might be produced by an abnormal enhancement of the interactions between the STN and GPe, with an oscillation frequency that depends on the excitatory cortical input to the STN and the inhibitory input to the GPe from the striatum [48–50]. Thus, the identification of the inhibitory pallido-cortical pathway might provide a structural basis for understanding the pathological motor features and local neural circuit regulation of the Parkinson’s disease. Similar to previous work [28, 29], it is interesting to use the current BGCT model to explore the detailed roles of this novel identified inhibitory pallido-cortical pathway in mediating between parkinsonian and normal states. In addition to the Parkinson’s disease, the GPe has also been reported to contribute to several high-level brain functions, especially the decision making [50, 51]. For example, recent studies have uncovered the activities of GPe could modulate decision threshold in the striatum, thus affecting the final results of decision making [51]. Theoretically, the direct pallido-cortical pathway may also participate in the regulation of the striatal threshold during decision making, but whether such opinion captures the real fact is still unclear. The BGCT model established in the present study might provide a modelling framework for investigating the underlying functional roles of GPe in several high-level brain functions.

Besides the indirect nigro-thalamic pathway relaying at superior colliculus that is not included in our current BGCT model, another possible model limitation is that the GPe neurons are not divided into subtypes according to their anatomical and firing proprieties [52–54]. As an important integrative hub for coordinating neuronal activity across the BG, the GPe is discovered to comprise a rich neural circuitry of diverse cell types [55–58]. In literature, two major groups of GPe neurons, termed the “prototypic” GPe neurons (PV-GPe) and the “arkypallidal” GPe neurons (Arky-GPe), were widely reported [53–58]. These two groups of GPe neurons make up more than 90% of all GPe neurons in rats and are responsible for communicating with different BG nuclei in anatomy, thus supporting different GPe-related functions [53–58]. However, due to the lack of detailed quantitative data, we considered all GPe neurons as a single neural population in our BGCT model. The similar method has been widely used in previous modelling studies [21, 28–30]. Such simplified modelling method allowed us to roughly evaluate the overall role of GPe in controlling absence seizures, but failed to discriminate the detailed roles contributed by different subtypes of GPe neurons. Further electrophysiological studies are needed to clarify the precise effects of these two groups of GPe neurons on the control of absence seizures.

Since absence epilepsy is a subtype of idiopathic generalized epilepsies, its dynamical activities are regarded as global brain activities [31–34]. Accordingly, some researchers have been proposed that different cortical areas, thalamic nuclei and their relevant neural projections might play different functional roles in the initiation, spreading and maintenance of 2–4 Hz SWDs during absence seizures. Despite that our developed BGCT model is powerful to inspire several functional roles of basal ganglia in controlling absence seizures, we have to admit that this biophysical model is idealized and cannot further identify the detailed contributions of different cortical areas, thalamic nuclei and neural projections to the initiation, spreading and maintenance of the typical 2–4 Hz SWDs. Fortunately, recent whole-brain computational modelling techniques using both the structural and functional magnetic resonance imaging data might provide us an approach to establish the large-scale BGCT model including multiple cortical areas and more complicated substructures in thalamus and basal ganglia [59–61]. By implementing such a large-scale BGCT model, it is possible to further investigate more detailed generation and regulation mechanisms of 2–4 Hz SWDs in our future work.

In summary, we have systematically performed computational studies to investigate the detailed roles of the novel identified GABAergic pallido-cortical pathway in controlling and modulating absence seizures. Our results demonstrated an intriguing hypothesis that the firing activities of GPe neurons indeed regulate the typical 2–4 Hz SWDs during absence seizures through this direct inhibitory pathway, indicating that the GPe might be an effective therapeutic target for treating absence seizures. Consistent with many previous experimental and modelling studies, our findings further emphasized the functional roles of BG in the control of absence seizures. In future studies, more direct electrophysiological evidence are required to experimentally validate the aforementioned hypothesis. Recent developments in optogenetic techniques, allowing selective activation/inactivation of specific groups of neurons, could be utilized to test the postulated control mechanism in a highly controlled manner [62, 63]. In addition, we expect that our computational results can inspire several underlying therapeutic strategies for real absence epileptic patients as well.

## Supporting Information

S1 Text The final first-order formulations for all neural populations in the modified BGCT model. (PDF)
