# Supplementary material for: Critical Roles of the Direct GABAergic Pallido-cortical Pathway in Controlling Absence Seizures
Source: PLoS Comput Biol. 2015 Oct 23;11(10):e1004539. doi: 10.1371/journal.pcbi.1004539 (PMC4619822; doi:10.1371/journal.pcbi.1004539)
Supplement: S1 Text — (PDF) [file pcbi.1004539.s001.pdf]

Critical Roles of the Direct GABAergic Pallido-cortical Pathway in Controlling Absence Seizures

Mingming Chen[1,*], Daqing Guo[1,2,*,†], Min Li[1], Tao Ma[1], Shengdun Wu[1],
Jingling Ma[1], Yan Cui[1], Yang Xia[1,2], Peng Xu[1,2], and Dezhong Yao[1,2,†]

[1]Key Laboratory for NeuroInformation of Ministry of Education, School of Life Science and Technology, University of Electronic Science and Technology of China, Chengdu 610054, People's Republic of China

[2]Center for Information in Medicine, University of Electronic Science and Technology of China, Chengdu 610054, People's Republic of China

June 10, 2015

S1 APPENDIX

By applying several reasonable assumptions mentioned in the main text, the modified BGCT model implemented in the present study becomes computationally more tractable without significant deteriorating the precision of numerical results. Then, the final mathematical description of our current BGCT model can be rewritten in the first-order, given as follows:

$$\frac{\mathrm{d}\phi_e(t)}{\mathrm{d}t} = \dot{\phi}_e(t), \tag{A1}$$

$$\frac{\mathrm{d}\dot{\phi}_e(t)}{\mathrm{d}t} = \gamma_e^2 \left\{-\phi_e(t) + F[V_e(t)]\right\} - 2\gamma_e\dot{\phi}_e(t), \tag{A2}$$

$$\frac{\mathrm{d}V_e(t)}{\mathrm{d}t} = \dot{V}_e(t), \tag{A3}$$

$$\frac{\mathrm{d}\dot{V}_e(t)}{\mathrm{d}t} = \alpha\beta \left\{-V_e(t) + v_{ee}\phi_e(t) + v_{ei}F[V_e(t)] + v_{ep_2}F[V_{p_2}(t)] + v_{es}F[V_s(t)]\right\} - (\alpha+\beta)\dot{V}_e(t), \tag{A4}$$

*These authors contributed to this work equally

†Corresponding authors: DY: dyao@uestc.edu.cn; DG: dqguo@uestc.edu.cn. Tel: +86-028-83201018, Fax: +86-028-83208238, Address: #4, Section 2, North JianShe Road, Chengdu 610054, People's Republic of China.

$$\frac{\mathrm{d}V_{d_1}(t)}{\mathrm{d}t} = \dot{V}_{d_1}(t), \tag{A5}$$

$$\frac{\mathrm{d}\dot{V}_{d_1}(t)}{\mathrm{d}t} = \alpha\beta\left\{-V_{d_1}(t) + v_{d_1e}\phi_e(t) + v_{d_1d_1}F[V_{d_1}(t)] + v_{d_1s}F[V_s(t)]\right\} - (\alpha+\beta)\dot{V}_{d_1}(t), \tag{A6}$$

$$\frac{\mathrm{d}V_{d_2}(t)}{\mathrm{d}t} = \dot{V}_{d_2}(t), \tag{A7}$$

$$\frac{\mathrm{d}\dot{V}_{d_2}(t)}{\mathrm{d}t} = \alpha\beta\left\{-V_{d_2}(t) + v_{d_2e}\phi_e(t) + v_{d_2d_2}F[V_{d_2}(t)] + v_{d_2s}F[V_s(t)]\right\} - (\alpha+\beta)\dot{V}_{d_2}(t), \tag{A8}$$

$$\frac{\mathrm{d}V_{p_1}(t)}{\mathrm{d}t} = \dot{V}_{p_1}(t), \tag{A9}$$

$$\frac{\mathrm{d}\dot{V}_{p_1}(t)}{\mathrm{d}t} = \alpha\beta\left\{-V_{p_1}(t) + v_{p_1d_1}F[V_{d_1}(t)] + v_{p_1p_2}F[V_{p_2}(t)] + v_{p_1\zeta}F[V_\zeta(t)]\right\} - (\alpha+\beta)\dot{V}_{p_1}(t), \tag{A10}$$

$$\frac{\mathrm{d}V_{p_2}(t)}{\mathrm{d}t} = \dot{V}_{p_2}(t), \tag{A11}$$

$$\frac{\mathrm{d}\dot{V}_{p_2}(t)}{\mathrm{d}t} = \alpha\beta\left\{-V_{p_2}(t) + v_{p_2d_2}F[V_{d_2}(t)] + v_{p_2p_2}F[V_{p_2}(t)] + v_{p_2\zeta}F[V_\zeta(t)]\right\} - (\alpha+\beta)\dot{V}_{p_2}(t), \tag{A12}$$

$$\frac{\mathrm{d}V_\zeta(t)}{\mathrm{d}t} = \dot{V}_\zeta(t), \tag{A13}$$

$$\frac{\mathrm{d}\dot{V}_\zeta(t)}{\mathrm{d}t} = \alpha\beta\left\{-V_\zeta(t) + v_{\zeta e}\phi_e(t) + v_{\zeta p_2}F[V_{p_2}(t)]\right\} - (\alpha+\beta)\dot{V}_\zeta(t), \tag{A14}$$

$$\frac{\mathrm{d}V_r(t)}{\mathrm{d}t} = \dot{V}_r(t), \tag{A15}$$

$$\frac{\mathrm{d}\dot{V}_r(t)}{\mathrm{d}t} = \alpha\beta\left\{-V_r(t) + v_{re}\phi_e(t) + v_{rp_1}F[V_{p_1}(t)] + v_{rs}F[V_s(t)]\right\} - (\alpha+\beta)\dot{V}_r(t), \tag{A16}$$

$$\frac{\mathrm{d}V_s(t)}{\mathrm{d}t} = \dot{V}_s(t), \tag{A17}$$

$$\begin{aligned}\frac{\mathrm{d}\dot{V}_s(t)}{\mathrm{d}t} =& \alpha\beta\left\{-V_s(t) + v_{se}\phi_e(t) + v_{sp_1}F[V_{p_1}(t)] + v_{sr}^{A}F[V_r(t)] + v_{sr}^{B}F[V_r(t-\tau)] + \phi_n\right\} \\ & -(\alpha+\beta)\dot{V}_s(t).\end{aligned} \tag{A18}$$
